# Phosphorylation of Na^+^,K^+^-ATPase at Tyr10 of the α1-Subunit is Suppressed by AMPK and Enhanced by Ouabain in Cultured Kidney Cells

**DOI:** 10.1007/s00232-021-00209-7

**Published:** 2021-11-08

**Authors:** Metka Petrič, Anja Vidović, Klemen Dolinar, Katarina Miš, Alexander V. Chibalin, Sergej Pirkmajer

**Affiliations:** 1 Faculty of Medicine, Institute of Pathophysiology, University of Ljubljana, Ljubljana, Slovenia; 2 National Research Tomsk State University, Tomsk, Russia; 3 Department of Molecular Medicine and Surgery, Integrative Physiology, Karolinska Institutet, Stockholm, Sweden

**Keywords:** AMPK, Ouabain, Tyr10, Na^+^, K^+^-ATPase, EGF, HK-2 cells

## Abstract

Na^+^,K^+^-ATPase (NKA) is essential for maintenance of cellular and whole-body water and ion homeostasis. In the kidney, a major site of ion transport, NKA consumes ~ 50% of ATP, indicating a tight coordination of NKA and energy metabolism. AMP-activated protein kinase (AMPK), a cellular energy sensor, regulates NKA by modulating serine phosphorylation of the α1-subunit, but whether it modulates other important regulatory phosphosites, such as Tyr10, is unknown. Using human kidney (HK-2) cells, we determined that the phosphorylation of Tyr10 was stimulated by the epidermal growth factor (EGF), which was opposed by inhibitors of Src kinases (PP2), tyrosine kinases (genistein), and EGF receptor (EGFR, gefitinib). AMPK activators AICAR and A-769662 suppressed the EGF-stimulated phosphorylation of EGFR (Tyr1173) and NKAα1 at Tyr10. The phosphorylation of Src (Tyr416) was unaltered by AICAR and increased by A-769662. Conversely, ouabain (100 nM), a pharmacological NKA inhibitor and a putative adrenocortical hormone, enhanced the EGF-stimulated Tyr10 phosphorylation without altering the phosphorylation of EGFR (Tyr1173) or Src (Tyr416). Ouabain (100–1000 nM) increased the ADP:ATP ratio, while it suppressed the lactate production and the oxygen consumption rate in a dose-dependent manner. Treatment with ouabain or gene silencing of NKAα1 or NKAα3 subunit did not activate AMPK. In summary, AMPK activators and ouabain had antagonistic effects on the phosphorylation of NKAα1 at Tyr10 in cultured HK-2 cells, which implicates a role for Tyr10 in coordinated regulation of NKA-mediated ion transport and energy metabolism.

**Graphical Abstract:**

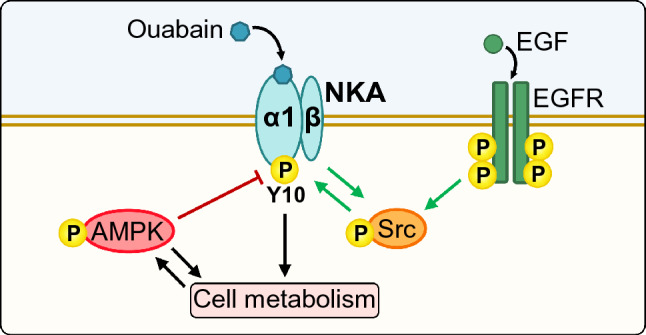

## Introduction

Na^+^,K^+^-ATPase (NKA), also known as Na–K pump, is essential for the cellular as well as the whole-body water and ion homeostasis (Rossier et al. 2015; Skou 1957, 1998). NKA, which comprises a catalytic α-subunit (isoforms α1–4) and a glycoprotein β-subunit (isoforms β1–3) (Blanco and Mercer 1998; Craig and Kyte 1980; Kyte 1971; Morth et al. 2007), consumes one ATP to pump two K^+^ ions into and three Na^+^ ions out of the cell (Post and Jolly 1957; Sen and Post 1964), thus maintaining ion concentrations, cell excitability, as well as the driving force for the Na^+^-driven secondary transport of nutrients, such as glucose. The abundance of NKA is especially high in the kidney (Buffin-Meyer et al. 1997; El Mernissi and Doucet 1984), which preserves the balance between the intake and loss of water and ions, while preventing urinary loss of glucose and other nutrients (Feraille and Dizin 2016; Feraille and Doucet 2001). The kidney consumes ~ 50% of ATP for energizing NKA (Clausen et al. 1991; Rolfe and Brown 1997), not least in the proximal tubules, which represent ~ 12% of the total ATP consumption in the kidney (Ferrannini 2017). In diabetes mellitus the proximal tubules reabsorb a markedly increased load of glucose and other solutes, which further increases ATP consumption by NKA (Ferrannini 2017). Clearly, a coordinated regulation of NKA and energy metabolism is required to maintain homeostasis.

**Fig. 1:**
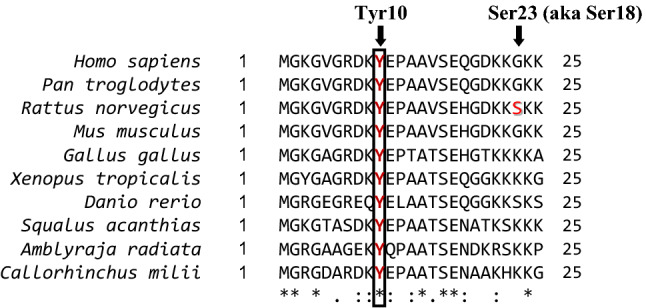
Tyr10 residue and surrounding amino acid sequence on the N terminus of α1-subunit of NKA are well-conserved in jawed vertebrates (Gnathostomata). The Ser23 (aka Ser18) residue is present in the rat NKA α1-subunit. Alignments of N terminus of α1-subunit of NKA among different species of Gnathostomata: *Homo sapiens* (human), *Pan troglodytes* (chimpanzee), *Rattus norvegicus* (brown rat), *Mus musculus* (house mouse), *Gallus gallus* (red junglefowl), *Danio rerio* (zebrafish), *Squalus acanthias* (spiny dogfish), *Amblyraja radiata* (thorny skate) and *Callorhinchus milii* (elephant shark) using Clustal Omega alignment program. Conservation scheme: [*] single, fully conserved residue, [:] strongly similar residues, [.] weakly similar residues

AMP-activated protein kinase (AMPK), a serine/threonine kinase, is a cellular energy sensor, important regulator of energy metabolism (Hardie 2018), and a leading experimental target for treatment of type 2 diabetes (Hardie 2013; Steinberg and Carling 2019), diabetic nephropathy, as well as polycystic kidney disease (Rajani et al. 2017). Once activated by an increase in the AMP:ATP and/or the ADP:ATP ratio, AMPK stimulates the ATP-generating catabolic and suppresses the ATP-consuming anabolic pathways, which helps to restore the energy balance under energy-deprived conditions. As well as the metabolic pathways, AMPK regulates phosphorylation of ion channels and transporters (Lang and Foller 2014; Rajani et al. 2017), including NKA (Pirkmajer et al. 2021), and therefore provides a direct link between regulation of ion transport and energy metabolism. AMPK regulates endocytosis of NKA by indirectly modulating the phosphorylation of the NKA α1-subunit (NKAα1) at Ser18 (aka Ser23) (Fig. 1) (Benziane et al. 2012). However, NKAα1 has other important phosphosites that might be modulated by AMPK.

Tyrosine phosphorylation of NKAα1 in the proximal kidney tubules is stimulated by insulin and epidermal growth factor (EGF) (Feraille et al. 1997). Among the phosphorylatable tyrosine residues, Tyr10 is particularly important not only because its phosphorylation is essential for the insulin-induced activation of NKA in the kidney tubules (Feraille et al. 1999), but also because it is conserved across the vertebrate species (Fig. 1). Aside from insulin, ouabain, a widely used pharmacological inhibitor of NKA and a putative adrenocortical hormone, was shown to increase phosphorylation of NKAα1 at Tyr10 in cultured kidney cells (Holthouser et al. 2010). As demonstrated in nonpigmented ciliary epithelial cells of the eye, the Tyr10 phosphorylation is increased also by nitric oxide (Shahidullah et al. 2014). Moreover, eplerenone, an antagonist of the aldosterone receptor, induced the dephosphorylation of Tyr10 in rat diaphragm muscle ex vivo (Breitenbach et al. 2016), which again suggests that Tyr10 plays an important role in hormonal regulation of NKA.

Tyrosine phosphorylation of NKAα is catalysed by the non-receptor tyrosine kinases from the Src family, such as Src (Al-Khalili et al. 2003) and Lyn (Bozulic et al. 2004a). Importantly, Src is a downstream effector of EGF receptor (EGFR) as well as ouabain. Binding of ouabain to NKA does not only inhibit its pumping but also activates multiple signalling pathways, including the EGFR-Src-ERK1/2 pathway (Aperia et al. 2016; Bagrov et al. 2009; Blaustein and Hamlyn 2020; Schoner and Scheiner-Bobis 2007; Xie and Askari 2002). Activation of Src might therefore provide a direct link between EGF, ouabain, and increased phosphorylation of Tyr10. Conversely, AMPK was shown to suppress the activation of the EGFR in cancer cells (Jhaveri et al. 2015), which indirectly suggests that AMPK may suppress the phosphorylation of Tyr10.

We examined whether and how EGF, AMPK, and ouabain modulated the phosphorylation of NKAα1 at Tyr10 in HK-2 cells, an epithelial cell line obtained from the proximal tubules of normal adult human kidney (Ryan et al. 1994). Our main finding was that pharmacological AMPK activators suppressed and ouabain enhanced the EGF-stimulated phosphorylation of Tyr10. Our results implicate a role for AMPK and ouabain in regulation of tyrosine phosphorylation of NKA.

## Materials and Methods

### Materials

Immortalized proximal tubule epithelial cell line HK-2 was from ATCC. Cell culture flasks and plates were from Sarstedt and TPP. Dulbecco's Modified Eagle Medium (DMEM), foetal bovine serum (FBS, No. 10270-106), Pen Strep (5000 U/mL of penicillin and 5000 µg/mL of streptomycin) and TaqMan Universal MasterMix and TaqMan gene expression assays were from Thermo Fisher Scientific. 4–12% Criterion XT Bis–Tris polyacrylamide gels and XT MES electrophoresis buffer were from Bio-Rad. PCR plates and PCR plate adhesive films were from Bio-Rad or Nippon Genetics. Amersham ECL Full-Range Rainbow Molecular Weight Markers were from GE Healthcare Life Sciences. Polyvinylidene difluoride (PVDF) membranes and all other reagents, unless specified otherwise, were from Merck.

### HK-2 Cell Cultures

HK-2 cell line was grown in DMEM GlutaMAX (1 g/L glucose, pyruvate) supplemented with 10% FBS and 1% Pen Strep at 37 °C in humidified air with 5% CO_2_ up until the passage 10. A day prior experiment the cells were seeded on 6-well cell culture plates at density of 2 × 10^5^ cells/well or on 12-well cell culture plates at density of 1 × 10^5^ cells/well (unless specified otherwise) to obtain 100% confluency at the time of experiment. For the last 4 h the cells were serum-starved in DMEM without serum and antibiotics.

### Treatments

The following substances were used for the treatment of the cells: activators of AMPK 5-aminoimidazole-4-carboxamide 1-β-d-ribofuranoside (AICAR; No. 10010241, Cayman Chemical) and A-769662 (No. 1466, Axon Medchem), mitochondrial oxidative phosphorylation uncoupler carbonyl cyanide-4-(trifluoromethoxy)phenylhydrazone (FCCP; No. C2920, Merck), inhibitor of Src kinases 3-(4-chlorophenyl)-1-(1,1-dimethylethyl)-1H-pyrazolo[3,4-d]pyrimidin-4-amine (PP2; No. 13198, Cayman Chemical), inhibitor of ATP synthase oligomycin (No. 495455, Merck), insulin (Actrapid, NovoNordisk), Na^+^,K^+^-ATPase inhibitor ouabain (No. O3125, Merck), epidermal growth factor (EGF; No. E9644, Merck), EGFR tyrosine kinase inhibitor gefitinib (No. 13166, Cayman Chemical), tyrosine kinase inhibitor genistein (No. 10005167, Cayman Chemical), electron transport chain complex I inhibitor rotenone (No. 557368, Merck), and complex III inhibitor antimycin A (No. A8674, Merck).

### Immunoblotting

Immunoblotting was performed as described (Dolinar et al. 2018; Mars et al. 2020; Pirkmajer et al. 2020). At the end of experiment, the cells were immediately transferred to ice and washed 2-times in ice-cold PBS (137 mM NaCl, 2.7 mM KCl, 10 mM Na_2_HPO_4_, 1.8 mM KH_2_PO_4_, pH 7.4). Cells were lysed with Laemmli buffer (62.5 mM Tris–HCl (pH 6.8), 2% (wt/vol) sodium dodecyl sulphate (SDS), 10% (wt/vol) glycerol, 5% (vol/vol) 2-mercaptoethanol, 0.002% (wt/vol) bromophenol blue) and were subsequently sonicated and heated at 56 °C for 20 min. Proteins were separated by SDS polyacrylamide gel electrophoresis with 4–12% gels and transferred to the PVDF membranes with wet electrotransfer. After the transfer, membranes were stained with Ponceau S [0.1% (wt/vol) in 5% (vol/vol) acetic acid] to evaluate uniformity of sample protein concentration. Membranes were then blocked in 7.5% (wt/vol) dry skimmed milk in Tris-buffered saline with Tween (TBST; 20 mM Tris, 150 mM NaCl, 0.02% (vol/vol) Tween 20, pH 7.6) for 1 h at room temperature, washed several times in TBST and then incubated with appropriate primary antibody (see Table 1) in primary antibody buffer (20 mM Tris, 150 mM NaCl, pH 7.6, 0.1% (wt/vol) bovine serum albumin and 0.1% (wt/vol) sodium azide) overnight at 4 °C. The next day membranes were washed in TBST and incubated with appropriate secondary antibody conjugated to the horseradish peroxidase (HRP, Bio-Rad) with 5% dry skimmed milk in TBST for 1 h at room temperature and washed with TBST. Finally, the membranes were incubated with chemiluminescence substrate (Pierce ECL Western Blotting Substrate, Thermo Fisher Scientific or, for weaker detection, Clarity Max ECL, Bio-Rad) for 1 min. The proteins were visualized on x-ray films (CP-BU NEW, AGFA HealthCare) which were scanned with GS-800 Densitometer (Bio-Rad) or imaged with FUSION FX6 (Vilber). Protein bands were quantified with Quantity One 1-D Analysis Software (4.6.9., Bio-Rad). Intensities of individual bands are presented relative to the total intensity of all the bands for each replicate.

**Table 1:** Antibodies used for immunoblot and immunoblot protocol details

Primary antibody	Species	Dilution	Cat. No	Secondary antibody	Dilution
Phospho-ACC (Ser79)	Rabbit pAb	1:1,000	#3661, CST	GAR	1:15,000
Total ACC	Rabbit mAb	1:1,000	#3676, CST	GAR	1:15,000
Actin	Rabbit pAb	1:1,500	#sc-1616-R, SCB	GAR	1:30,000
Phospho-Akt (Ser473)	Rabbit mAb	1:2,000	#4060, CST	GAR	1:15,000
Phospho-AMPKα (Thr172)	Rabbit mAb	1:1,000	#2535, CST	GAR	1:10,000
Caspase-3	Rabbit pAb	1:1,000	#9662, CST	GAR	1:20,000
Phospho-EGFR (Tyr1173)	Rabbit mAb	1:1,000	#4407, CST	GAR	1:10,000
Total EGFR	Rabbit pAb	1:1,000	#2232, CST	GAR	1:20,000
Phospho-ERK1/2 (Thr202/Tyr204)	Rabbit mAb	1:20,000	#4370, CST	GAR	1:20,000
Total ERK1/2	Rabbit mAb	1:1,000	#4695, CST	GAR	1:20,000
Phospho-NKAα1 (Tyr10) mAb	Rabbit mAb	1:500	#13566, CST	GAR	1:10,000
Phospho-NKAα1 (Tyr10) pAb	Rabbit pAb	1:1,000	#3060, CST	GAR	1:10,000
Total NKAα1 (Ab1)	Mouse mAb	1:2,000	#05–369, Merck	GAM	1:25,000
Total NKAα1 (Ab2)	Rabbit mAb	1:1,000	#23565, CST	GAR	1:20,000
Phospho-Src (Tyr416)	Rabbit pAb	1:1,000	#2101, CST	GAR	1:10,000
Phospho-Src (Tyr527)	Rabbit pAb	1:1,000	#2105, CST	GAR	1:15,000
Total Src	Rabbit mAb	1:1,000	#2123, CST	GAR	1:25,000

*ACC* acetyl-CoA carboxylase, *Akt* protein kinase B, *AMPK* AMP-activated protein kinase, *EGFR* epidermal growth factor receptor, *ERK1/2* p44/42 mitogen-activated protein kinases (MAPK), *NKA* Na^+^,K^+^-ATPase, *pAb* polyclonal antibody, *mAb* monoclonal antibody, *CST* Cell Signaling Technology, *SCB* Santa Cruz Biotechnology, *GAR* goat anti-rabbit IgG-HRP conjugate #1706515 (Bio-Rad), *GAM* goat anti-mouse IgG-HRP conjugate #1706516 (Bio-Rad)

### Gene Silencing

HK-2 cells were seeded on 6-well cell culture plates at density of 1 × 10^5^ cells/well a day prior transfection to achieve 80% confluency at the time of transfection. For the last 6 h the cells were grown in medium without antibiotics. The cells were transfected with siRNA against human Na^+^,K^+^-ATPase α1 mRNA (ATP1A1, No. L-006111-00-0005) or human Na^+^,K^+^-ATPase α3 mRNA (ATP1A3, No. J-004614-00-0005) (both ON-TARGETplus–SMARTpool from Horizon Discovery) using Invitrogen Lipofectamine 2000 Transfection Reagent (Thermo Fisher Scientific) according to the manufacturer’s guidelines with siRNA in the final concentration of 5 nM. To test the off-target effects of siRNA, a non-targeting siRNA pool (ON-TARGET plus Non-targeting Pool, No. D-001810-10-20, Horizon Discovery) was used. After 24 h of transfection the medium was replaced with fresh DMEM (10% FBS, without antibiotics/siRNA) and the cells were incubated for another 24 h. To enable measurement of lactate secretion into medium, the cells were grown in serum-free DMEM for the last 8 h. The cells were washed with PBS and frozen at − 80 °C until further analysis. Gene knock-down was assayed using qPCR and immunoblot.

### qPCR

Total RNA was isolated using RNeasy Plus Mini Kit (Qiagen) or E.Z.N.A. HP Total RNA Kit (Omega Bio-tek) and reverse transcribed to cDNA with High-Capacity cDNA Reverse Transcription Kit (Thermo Fisher Scientific). Quantitative real-time polymerase chain reaction (qPCR) was performed on 7500 Real-Time PCR System (Applied Biosystems, Thermo Fisher Scientific) using TaqMan Universal MasterMix and TaqMan gene expression assays for human α1-subunit of Na^+^,K^+^-ATPase (No. Hs00167556_m1) and human α3-subunit of Na^+^,K^+^-ATPase (No. Hs00958036_m1). Expression of target gene was normalized to expression of cyclophilin (PPIA, No. Hs99999904_m1), which was used as an endogenous control. Efficiency of PCR was estimated with LinRegPCR software (2018.0) (Ramakers et al. 2003; Ruijter et al. 2009; Tuomi et al. 2010).

### Analysis of Extracellular Acidification Rate (ECAR) and Oxygen Consumption Rate (OCR)

ECAR and OCR were analysed with Seahorse XFe24 Analyzer (Agilent). Cells were seeded on Seahorse XF24 cell culture microplates (Agilent) at density of 50 × 10^3^ cells/well. 24 h after seeding, growth medium was replaced with Seahorse XF DMEM medium (Agilent) supplemented with 10 mM glucose, 1 mM pyruvate and 2 mM glutamine. Cells were incubated for 1 h at 37 °C in normal atmosphere (no additional CO_2_) and then transferred into Seahorse. In Seahorse, cells were treated with ouabain or vehicle for ~ 3½ h, which was followed by successive treatment with 1 μM oligomycin, 0.75 μM FCCP, and a combination of 1 μM rotenone and 1 μM antimycin A.

### Analysis of Cell Death with Flow Cytometry

At the end of the experiment cells were detached with trypsinization and stained with FITC-conjugated annexin V (for detection of phosphatidylserine on the outer membrane of apoptotic cells) and propidium iodide (PI; for detection of cells with permeable cell membrane) using Annexin V FITC Assay Kit (No. 600300, Cayman Chemical). Cells were analysed with Cell Lab Quanta SC flow cytometer (Beckman Coulter) and divided into four populations based on the intensity of FITC and PI fluorescence: live (low FITC, low PI), early apoptotic (high FITC, low PI), late apoptotic (high FITC, high PI), and necrotic (low FITC, high PI).

### 2-Deoxy-Glucose (2-DG) Uptake Assay

Glucose uptake was determined by measuring the uptake of tritium (^3^H)-labelled 2-deoxy-glucose (2-DG), as described (Dolinar et al. 2018; Pirkmajer et al. 2015). Cells were washed with HEPES-buffered saline (HBS: 140 mM NaCl, 20 mM HEPES, 5 mM KCl, 2.5 mM MgCl_2_, 1 mM CaCl_2_, pH 7.4) and incubated in HBS with 10 μM 2-DG and 1 μCi/mL 2-[1,2-^3^H]-DG (PerkinElmer) for 10 min at 37 °C. Cells were subsequently washed with PBS supplemented with 25 mM glucose and lysed with 0.04% (wt/vol) SDS in water. Cell lysates were then analysed for protein content with Pierce BCA Protein Assay Kit (Thermo Fisher Scientific) or mixed with liquid scintillation cocktail Aquasol 2 (PerkinElmer) and analysed for radioactivity with MicroBeta TriLux scintillation counter (PerkinElmer). Amount of 2-DG taken up by the cells was determined with the help of a standard (HBS with 10 μM 2-DG and 1 μCi/mL 2-[1,2-^3^H]-DG) and expressed in pmol/min/mg of protein. Control cells were treated with inhibitors of glucose transporters (phlorizin (100 μM) or cytochalasin B (10 μM)) during 2-DG uptake to confirm that 2-DG was taken up via glucose transporters.

### Lactate Dehydrogenase (LDH), Lactate, Protein and DNA Assays

Lactate dehydrogenase (LDH) and lactate in cell medium were measured with Cytotoxicity Detection Kit (LDH) (Roche) and Lactate Assay Kit (Merck), respectively. The total protein content was determined with Pierce BCA Protein Assay Kit (Thermo Fisher Scientific). All assays were performed according to the manufacturer’s instructions. The DNA content was determined with a DNA assay based on the fluorescent DNA dye Hoechst 33342 (Thermo Fisher Scientific) as described (Rajh et al. 2016). Briefly, after lysis with 0.04% (wt/vol) SDS in water cell lysates were transferred to a 96-well microplate, diluted to 100 μL with water and mixed with 100 μL of Tris-NaCl buffer (50 mM Tris, 100 mM NaCl, pH 8.3) with 10 μg/mL Hoechst 33342. Samples were incubated for 15 min at room temperature and then Hoechst fluorescence was measured with VICTOR microplate reader (PerkinElmer) using 355 nm excitation filter and 460 nm emission filter.

### ADP/ATP Ratio Assay

The ADP/ATP ratio was determined with ADP/ATP Ratio Assay Kit (Merck) according to the manufacturer’s instructions. Cells were seeded in white 96-well microplates with clear bottom (PerkinElmer). Luminescence was measured with VICTOR microplate reader.

### Statistical Analysis

Results are presented as means with standard error of the mean (± SEM). Results were statistically analysed with GraphPad Prism 6 software using one-way ANOVA followed by Bonferroni’s or Dunnett's post hoc test to compare different treatments and control samples or Student's t-test to compare only two samples. *P* ≤ 0.05 was considered as statistically significant.

## Results

### A Validation of the Primary Antibodies Against NKAα1 and Phospho-NKAα1 (Tyr10)

**Fig. 2:**
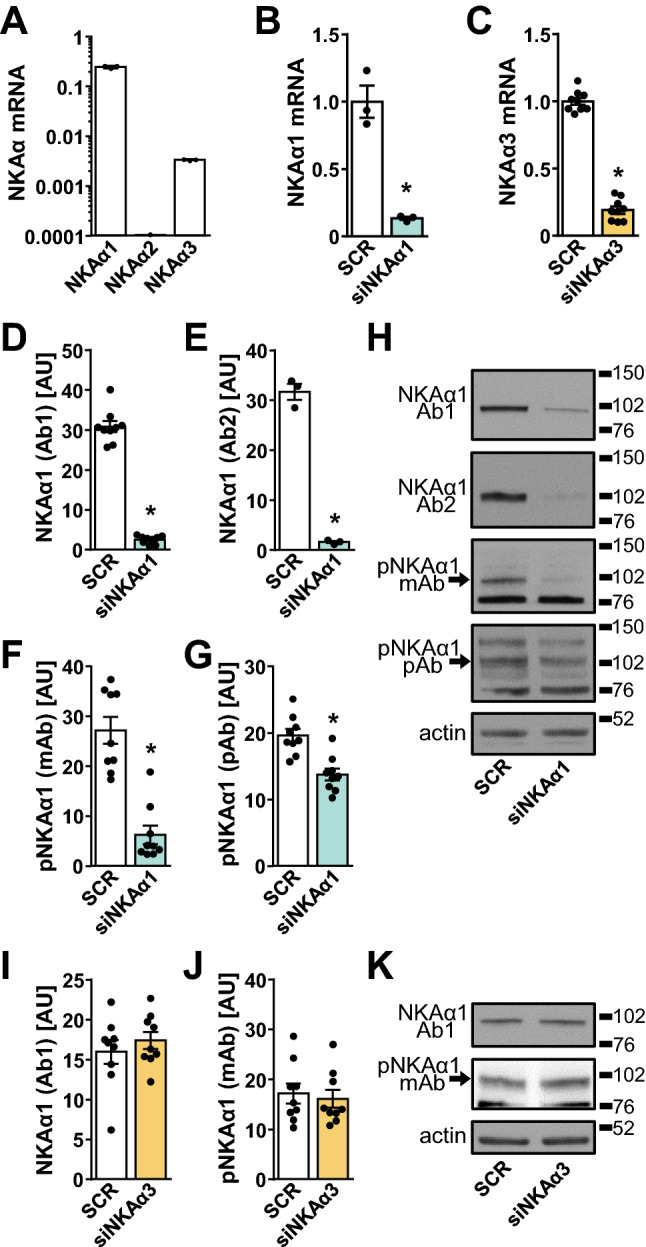
Validation of primary antibodies against NKAα1 and phospho-NKAα1 (Tyr10). **A** The mRNA expression of NKAα isoforms in HK-2 cells reported as gene expression ratio. The endogenous control was cyclophilin A (PPIA). Results are means with SEM, *n* = 3 (one experiment). **B**–**K** Gene silencing of NKAα1 (**B**, **D**–**H**) or NKAα3 (**C**, **I**–**K**) in HK-2 cells was performed to evaluate the specificity of primary antibodies against NKAα1 and phospho-NKAα1 (Tyr10) (pNKAα1). Gene expression and protein abundance were measured 48 h after exposure to 5 nM scrambled siRNA (SCR) or siRNA against NKAα1 (siNKAα1) or NKAα3 (siNKAα3). qPCR was performed to estimate NKAα1 (**B**) and NKAα3 (**C**) mRNA expression. Results are reported as fold-change relative to the SCR-treated cells. The endogenous control was cyclophilin A (PPIA), *n* = 3 or 9 (one or three experiments). Immunoblotting was used to estimate the abundance of the total NKAα1 (**D**, **E**, **I**) and phospho-NKAα1 (Tyr10) (**F**, **G**, **J**) using four different antibodies: mAb #05-369 (Ab1) (**D**, **I**) or mAb #23566 (Ab2) for NKAα1 (**E**) and mAb #13566 (**F**, **J**) or pAb #3060 (**G**) for pNKAα1 (Tyr10). Representative blots of silencing of NKAα1 are shown in panel **H** and of NKAα3 in panel **K**. Numbers next to the blots indicate molecular weight markers (kDa). Results of immunoblotting are presented as means with SEM, three independent experiments, *n* = 9, except in panel **E** (one experiment, *n* = 3). *P < 0.05 vs. SCR (Student’s *t*-test)

NKAα1 is the predominant NKAα isoform in HK-2 cells; however, they also express NKAα3 (Fig. 2A), with which the antibodies against the NKAα1 isoform might cross-react. To test this possibility, NKAα1 (Fig. 2B) or NKAα3 (Fig. 2C) were knocked-down by siRNA. Gene silencing of NKAα1 markedly reduced the intensity of the immunoreactive band at the ~ 102 kDa molecular weight marker, which was detected with two different (Ab1 and Ab2) anti-NKAα1 primary antibodies (Fig. 2D,E,H). The phosphorylation of Tyr10 of NKAα1 was also detected with two (mAb and pAb) different primary antibodies (Fig. 2F–H). In addition to the ~ 102 kDa band, which was sensitive to gene silencing of NKAα1, these antibodies detected another band at the ~ 76 kDa molecular weight marker (Fig. 2F–H), whose intensity was the same in control and NKAα1-deficient cells. Gene silencing of NKAα3 had no effect on the detection of the total NKAα1 or the phosphorylated NKAα1 (Tyr10) (Fig. 2I-K).

Taken together with extremely low expression of NKAα2 mRNA (Fig. 2A), these results suggested that these primary antibodies were suitable for detection of the total or phosphorylated NKAα1 in HK-2 cells. All further analyses were carried out with the monoclonal antibody (Ab1) against the total NKAα1 and the monoclonal antibody (mAb) against the phosphorylated NKAα1 (Tyr10). The details about the antibodies are presented in Table 1.

### Effect of Insulin and EGF on the Phosphorylation of NKAα1 (Tyr10)

**Fig. 3:**
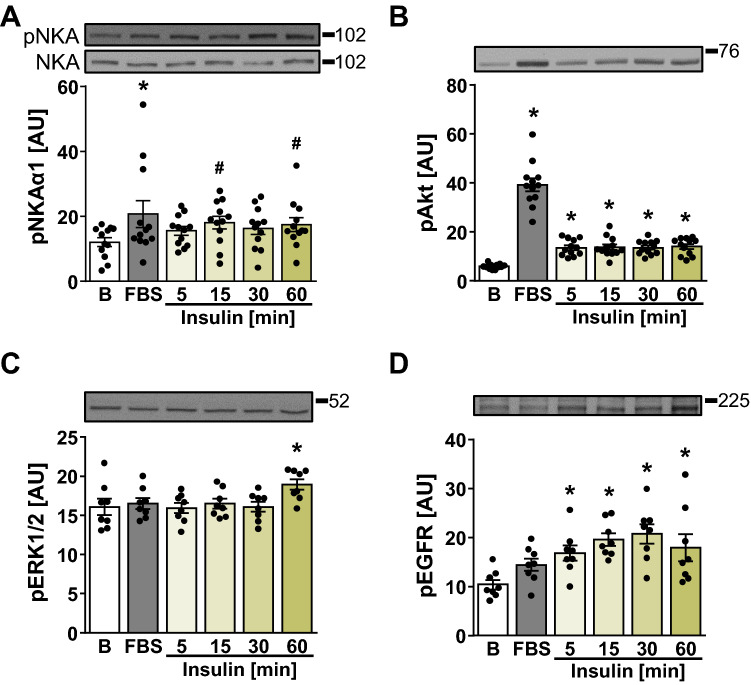
Time-dependent effects of insulin on NKAα1 phosphorylation and on the associated signalling pathways in HK-2 cells. **A**–**D** HK-2 cells were treated with 10% FBS for 30 min or with 120 nM insulin for 5–60 min as indicated. Immunoblotting was used to estimate protein levels of phospho-NKAα1 (Tyr10) (**A**), phospho-Akt (Ser473) (**B**), phospho-ERK1/2 (Thr202/Tyr204) (**C**), phospho-EGFR (Tyr1173) (**D**). Numbers next to the blots indicate molecular weight markers (kDa), **B** basal. Results are means with SEM (three independent experiments, *n* = 12). *P < 0.05 vs. basal (unpaired one-way ANOVA, Dunnett’s test), ^#^*P* < 0.05 vs. basal (Student’s *t*-test)

HK-2 cells were treated with 10% FBS (30 min) or 120 nM insulin (5–60 min) (Fig. 3). FBS increased the phosphorylation of NKAα1 (Tyr10) although its effect was variable (Fig. 3A), possibly due to the use of different batches. Insulin did not increase the phosphorylation of NKAα1 (Tyr10) significantly as assessed with Dunnett’s post hoc test (Fig. 3A), although phosphorylation tended to be increased in the insulin-treated samples at all time points. The most pronounced increase was detected with the 15-min insulin treatment, which was significant with Student’s *t*-test. Due to a modest response of Tyr10 to the stimulation with insulin, the signalling pathways downstream of the insulin receptor were also assessed. Insulin increased the phosphorylation of Akt (Ser473) at all time points (Fig. 3B) and the phosphorylation of ERK1/2 (Thr202/Tyr204) at 60 min (Fig. 3C), indicating HK-2 cells were responsive to the insulin treatment. The phosphorylation of EGFR (Tyr1173) was increased by insulin during the entire time course (Fig. 3D). These results indicated that while phosphorylation of Tyr10 in HK-2 cells is responsive to exogenous stimuli, insulin was not a strong stimulus.

**Fig. 4:**
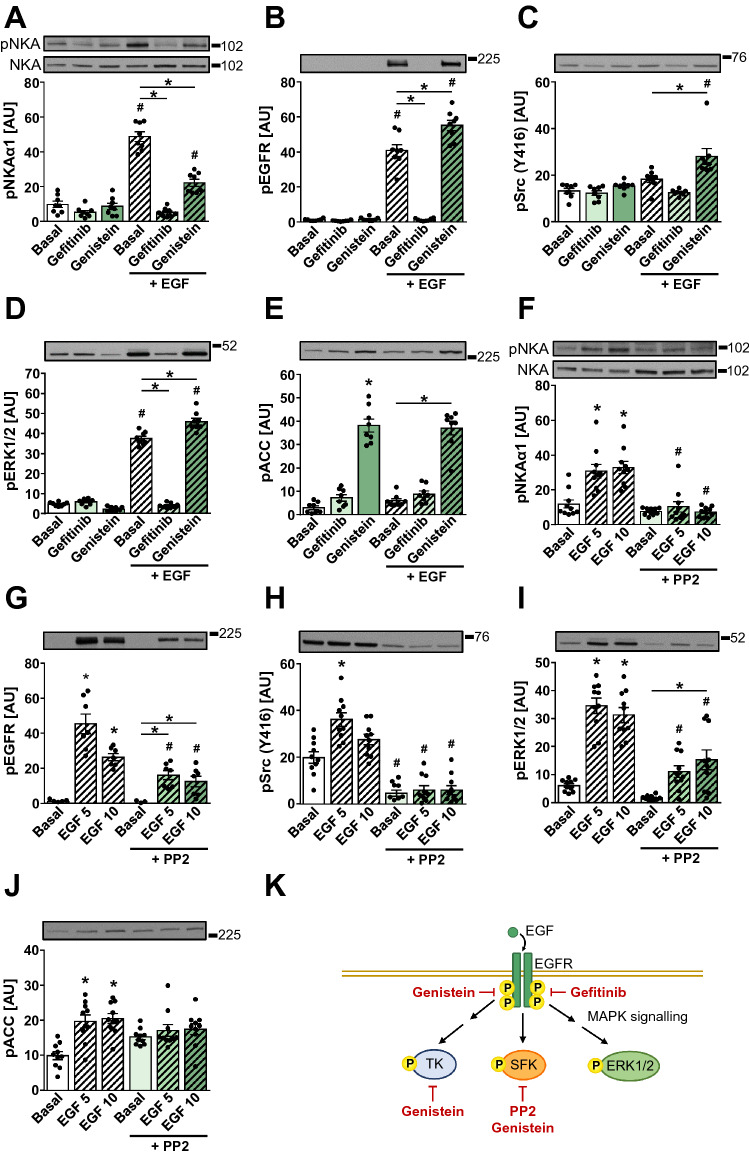
EGF regulates Tyr10 phosphorylation of NKAα1 in HK-2 cells via the EGFR-Src pathway. **A**–**E** HK-2 cells were pretreated with or without 10 µM EGFR inhibitor gefitinib or 100 µM tyrosine kinase inhibitor genistein for 60 min and stimulated with 1 µg/mL EGF during the last 10 min (two independent experiments, *n* = 8). **F**–**J** HK-2 cells were pretreated with or without 20 µM Src family kinase inhibitor PP2 for 25 min and stimulated with 1 µg/mL EGF during the final 5 or 10 min. Results are means with SEM (three independent experiments, *n* = 10). Immunoblotting was used to estimate the abundance of phospho-NKAα1 (Tyr10) (**A**, **F**), phospho-EGFR (Tyr1173) (**B**, **G**), phospho-Src (Tyr416) (**C**, **H**), phospho-ERK1/2 (Thr202/Tyr204) (**D**, **I**), and phospho-ACC (Ser79) (**E**, **J**). Numbers next to the blots indicate molecular weight markers (kDa). **K** Schematic presentation of signalling pathways and site of action of inhibitors used in this study. *SFK* Src family kinases, *TK* tyrosine kinases. *P < 0.05 vs. basal, ^#^*P* < 0.05 EGF/PP2 vs. no EGF/PP2 (unpaired one-way ANOVA, Bonferroni’s test)

To further explore the signalling pathways that link the extracellular signals to the phosphorylation of NKAα1 (Tyr10), HK-2 cells were treated with 1 μg/mL EGF (10 min) in the presence of EGFR inhibitor gefitinib (10 μM) or tyrosine kinase inhibitor genistein (100 μM) (Akiyama et al. 1987; Ogawara et al. 1986) (Fig. 4K). EGF markedly increased the phosphorylation of NKAα1 at Tyr10 (Fig. 4A). The increase in Tyr10 phosphorylation was prevented by gefitinib and blunted by genistein (Fig. 4A). This was paralleled by a complete suppression of the EGF-stimulated phosphorylation of EGFR (Fig. 4B) and ERK1/2 (Fig. 4D) by gefitinib. Genistein increased the activating phosphorylation of Src (Tyr416) (Fig. 4C), but had no effect on the EGFR or ERK1/2 phosphorylation in the EGF-treated HK-2 cells. We also measured the phosphorylation of acetyl-CoA carboxylase (ACC) at Ser79 (Fig. 4E), an AMPK substrate and an indicator of AMPK activation (Carling et al. 1987; Davies et al. 1990), which was markedly increased by genistein. These results showed that the EGF-stimulated phosphorylation of Tyr10 is completely suppressed by the EGFR inhibition and blunted by a non-specific inhibition of tyrosine kinases.

Src was shown to increase tyrosine phosphorylation of NKAα under in vitro conditions (Al-Khalili et al. 2003). Since Src is downstream of EGFR, we examined whether Src is required for the EGF-stimulated phosphorylation of Tyr10. HK-2 cells were treated with 1 μg/mL EGF for 5 or 10 min in the presence or absence of PP2, an inhibitor of Src family of protein kinases. EGF stimulated the phosphorylation of NKAα1 (Tyr10) (Fig. 4F), EGFR (Fig. 4G), Src (Fig. 4H), as well as ERK1/2 (Fig. 4I). PP2 markedly suppressed the phosphorylation of Src in the absence or presence of EGF (Fig. 4H) and prevented the EGF-stimulated increase in NKAα1 (Tyr10) phosphorylation (Fig. 4F). PP2 blunted but did not prevent the EGF-stimulated phosphorylation of EGFR and ERK1/2 (Fig. 4G, I). The phosphorylation of ACC, a substrate of AMPK, was somewhat increased by EGF (Fig. 4J). Taken together, these results suggested that EGF stimulated the phosphorylation of NKAα1 (Tyr10) via the EGFR-Src pathway.

### AMPK Suppresses EGF-Stimulated Phosphorylation of NKAα1 in HK-2 Cells

**Fig. 5:**
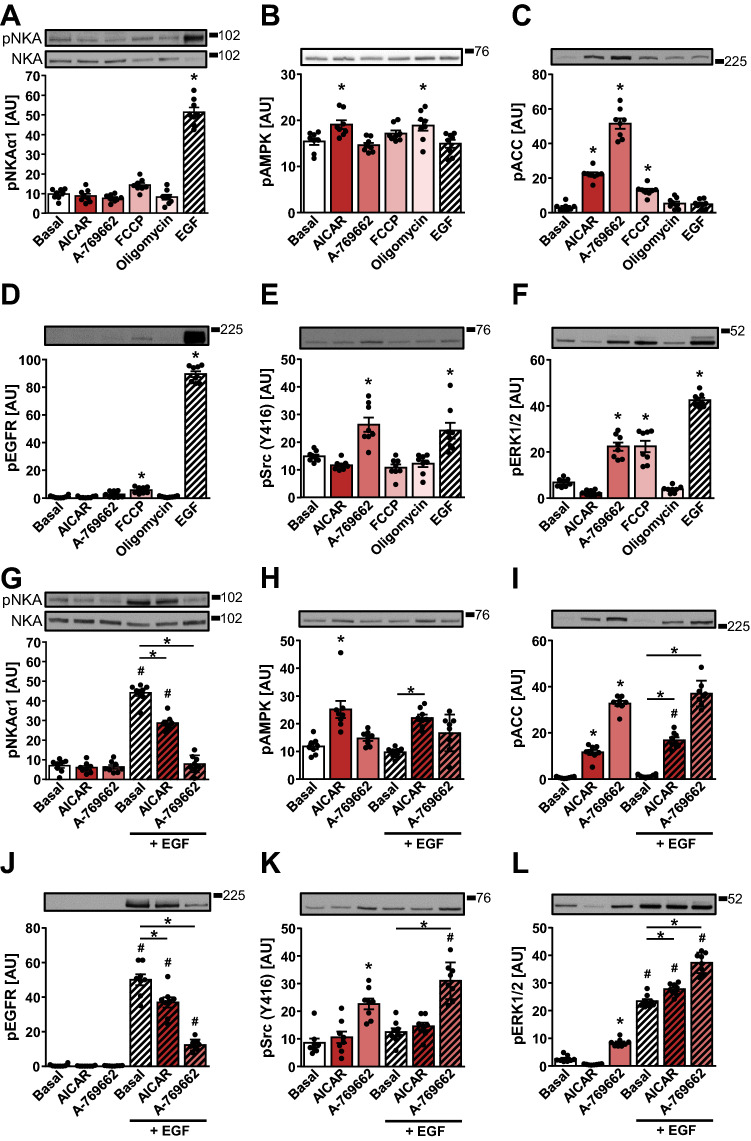
AMPK suppresses EGF-stimulated Tyr10 phosphorylation in HK-2 cells. **A**–**F** HK-2 cells were treated with direct and indirect AMPK activators for 60 min: 2 mM AICAR, 300 µM A-769662, 10 mM 2-deoxy-glucose (2-DG, glycolysis inhibitor), 30 µM FCCP (mitochondrial uncoupler) or 1 µM oligomycin (inhibitor of ATP synthase). Stimulation with 1 μg/mL EGF for 10 min was used as a positive control of Tyr10 phosphorylation of NKA. **G**–**L** HK-2 cells were treated with 2 mM AICAR or 300 µM A-769662 for 60 min. During the last 10 min HK-2 cells were stimulated with 1 µg/mL EGF. Immunoblotting was used to estimate protein levels of phospho-NKAα1 (Tyr10) (**A**, **G**), phospho-AMPK (Thr172) (**B**, **H**), phospho-ACC (Ser79) (**C**, **I**), phospho-EGFR (Tyr1173) (**D**, **J**), phospho-Src (Tyr416) (**E**, **K**) and phospho-ERK1/2 (Thr202/Tyr204) (**F**, **L**). Numbers next to the blots indicate molecular weight markers (kDa). Results are means with SEM (two independent experiments, *n* = 8). **A–F** *P < 0.05 vs. basal (unpaired one-way ANOVA, Dunnett´s test). **G**–**L** *P < 0.05 vs. basal, ^#^*P* < 0.05 EGF vs. no EGF (unpaired one-way ANOVA, Bonferroni’s test)

Pharmacological inhibition of EGFR/HER2 was shown to activate AMPK in cancer cells (Spector et al. 2007), indirectly suggesting that EGFR and AMPK may exert functionally opposing effects on the Tyr10 phosphorylation. HK-2 cells were treated with AICAR (Corton et al. 1995; Sullivan et al. 1994) and A-769662 (Cool et al. 2006), which bind to AMPK and activate it directly, and mitochondrial uncoupler FCCP and inhibitor of ATP synthase oligomycin, which activate AMPK by suppressing the ATP synthesis (Hawley et al. 2010; Rohas et al. 2007). The phosphorylation of Tyr10 was markedly increased by EGF, while AMPK activators did not alter it (Fig. 5A). The activating phosphorylation of AMPK at Thr172 was slightly increased by AICAR and oligomycin (Fig. 5B), while the phosphorylation of ACC was markedly increased by AICAR, A-769662, and FCCP (Fig. 5C), indicating AMPK activation. FCCP increased the phosphorylation of EGFR (Fig. 5D) and ERK1/2 (Fig. 5F), while A-769662 increased the phosphorylation of Src (Fig. 5E) and ERK1/2 (Fig. 5F).

These results did not support the notion that AMPK might modulate the phosphorylation of Tyr10. However, under basal conditions the phosphorylation of EGFR was low (Figs. 4G and 5D), while gefitinib (Fig. 4A) and PP2 (Fig. 4F) tended to have only a marginal effect on basal phosphorylation of Tyr10, indirectly suggesting that the EGFR-Src pathway may not be an important regulator of Tyr10 under non-stimulated conditions. To examine this possibility HK-2 cells were treated with EGF after being pretreated with AMPK activators AICAR and A-769662 (Fig. 5G–L). The EGF-stimulated phosphorylation of Tyr10 was suppressed by AICAR and abolished by A-769662 (Fig. 5G). Although only AICAR increased the phosphorylation of AMPK (Fig. 4H), a marked increase in the ACC phosphorylation (Fig. 5I) suggested potent AMPK activation by AICAR and A-769662. AICAR and A-769662 both suppressed the EGF-stimulated EGFR phosphorylation (Fig. 5J), but they showed divergent effects on the phosphorylation of Src. Unlike AICAR, A-769662 potently increased the Src phosphorylation at Tyr416 with or without EGF (Fig. 5E, K). Similarly, while AICAR suppressed the phosphorylation of ERK1/2 in the absence of EGF, A-769662 stimulated it (Fig. 5L). Taken together, these results suggested that pharmacological AMPK activation opposed the EGF-stimulated phosphorylation of EGFR (Tyr1173) as well as NKAα1 (Tyr10), although AICAR and A-769662 produced divergent downstream signalling events. Further, they showed that activation of Src, as assessed by phosphorylation of Tyr416, per se was not sufficient to stimulate the phosphorylation of Tyr10.

### Ouabain Enhances the EGF-Stimulated Phosphorylation of NKAα1 (Tyr10) in HK-2 Cells

**Fig. 6:**
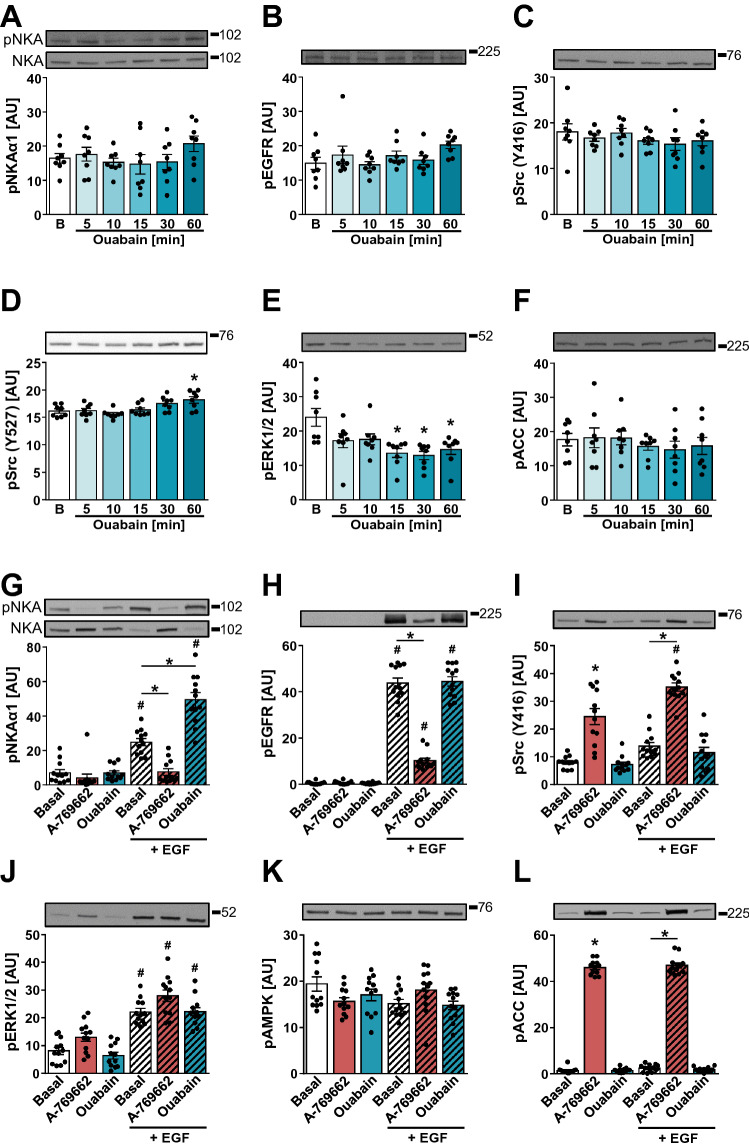
Ouabain enhances EGF-stimulated Tyr10 phosphorylation in HK-2 cells. **A**–**F** HK-2 cells were treated with 100 nM ouabain for 5, 10, 15, 30 or 60 min. **G**–**L** HK-2 cells were treated with 300 µM A-769662 or 100 nM ouabain for 60 min. 1 µg/mL EGF was added during the last 10 min. Immunoblotting was used to estimate the abundance of phospho-NKAα1 (Tyr10) (**A**, **G**), phospho-EGFR (Tyr1173) (**B**, **H**), phospho-Src (Tyr416) (**C**, **I**), phospho-Src (Tyr527) (**D**), phospho-ERK1/2 (Thr202/Tyr204) (**E**, **J**), phospho-ACC (Ser79) (**F**, **L**), and phospho-AMPK (Thr172) (**K**). Numbers next to the blots indicate molecular weight markers (kDa), **B** basal. Results are means with SEM (two or three independent experiments, *n* = 8–12). **A**–**F** *P < 0.05 vs. basal (unpaired one-way ANOVA, Dunnett´s test). **G**–**L** *P < 0.05 vs. basal, ^#^*P* < 0.05 EGF vs. no EGF (unpaired one-way ANOVA, Bonferroni’s test)

To explore the possibility that ouabain affects the phosphorylation of Tyr10 via Src, HK-2 cells were treated with 100 nM ouabain. The treatment with ouabain (5–60 min) did not alter the phosphorylation of NKAα1 at Tyr10 (Fig. 6A), EGFR (Fig. 6B), and Src (Tyr416) (Fig. 6C). The inhibitory phosphorylation of Src at Tyr527 was increased at 60 min (Fig. 6D), while the phosphorylation of ERK1/2 was suppressed between 15–60 min of the ouabain treatment (Fig. 6E). The phosphorylation of ACC was unaltered (Fig. 6F).

HK-2 cells were then treated with 1 μg/mL EGF (10 min) in the absence or presence of 100 nM ouabain or 300 μM A-769662 (Fig. 6G–L). Ouabain enhanced the EGF-stimulated phosphorylation of NKAα1 (Tyr10) (Fig. 6G), while it did not affect the phosphorylation of EGFR (Fig. 6H) or Src (Tyr416) (Fig. 6I). AMPK activator A-769662 again suppressed the EGF-stimulated phosphorylation of Tyr10 (Fig. 6G) and EGFR (Fig. 6H), while enhancing the EGF-stimulated phosphorylation of Src (Tyr416) (Fig. 6I). The phosphorylation of ERK1/2 was unaltered by ouabain, while it was increased by A-769662 (Fig. 6J). Ouabain did not modulate the phosphorylation of ERK1/2, AMPK, and/or ACC (Fig. 6J–L). Taken together, these results showed that ouabain markedly enhanced the EGF-stimulated phosphorylation of Tyr10 without enhancing the phosphorylation of EGFR (Tyr1173) or Src (Tyr416).

### The Role of NKAα1 and NKAα3 in Regulation of Intracellular Signalling in HK-2 Cells Under Basal Conditions

**Fig. 7:**
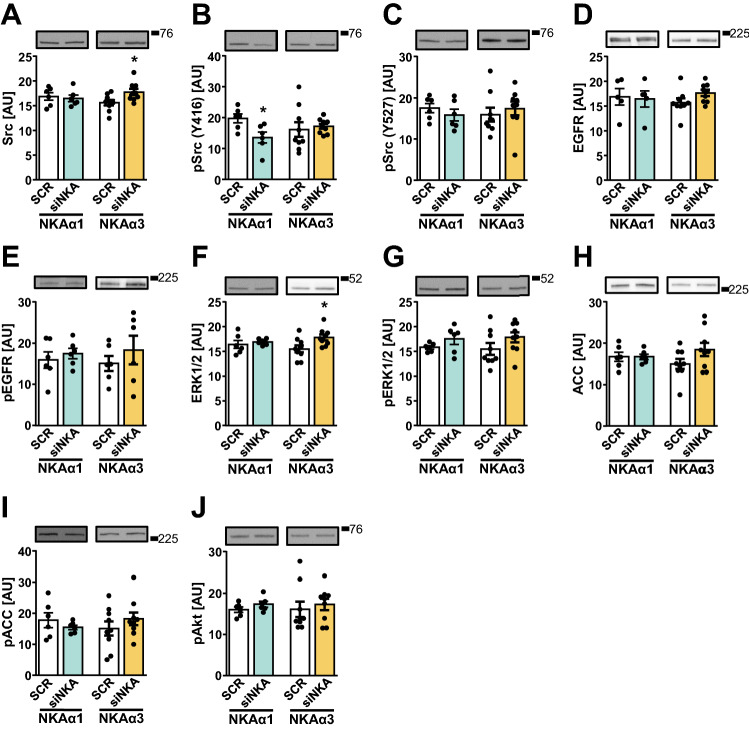
The effects of silencing of NKAα1 and NKAα3 on NKA-associated signalling pathways in HK-2 cells. The effect of silencing of α1 (left parts of graphs) and α3-subunit (right parts of graphs) of NKA on phospho and total protein levels of Src, EGFR, Erk1/2, ACC, and Akt. The cells were treated with 5 nM scrambled siRNA (SCR) or siRNA against NKAα1 or NKAα3 and cultured for 48 h. For the last 8 h the cells were grown in DMEM w/o serum. Immunoblot was used to evaluate the effects of silencing on protein levels of Src (**A**), phospho-Src (Tyr416) (**B**), phospho-Src (Tyr527) (**C**), EGFR (**D**), phospho-EGFR (Tyr1173) (**E**), ERK1/2 (**F**), phospho-ERK1/2 (Thr202/Tyr204) (**G**), ACC (**H**), phospho-ACC (Ser79) (**I**) and phospho-Akt (Ser473) (**J**). Numbers next to the blots indicate molecular weight markers (kDa). Results are mean with SEM (two or three independent experiments, *n* = 6–9). *P < 0.05 vs. SCR (Student’s *t*-test)

Ouabain had only a minor effect on intracellular signalling pathways under basal conditions (Fig. 6). To further explore the link between NKA and signalling in HK-2 cells, we measured the abundance of multiple phosphoproteins involved in signalling via EGFR and insulin receptor after knocking-down NKAα1 or NKAα3 with siRNA (Fig. 7). The knock-down of NKAα1 suppressed the level of the phosphorylated Src (Tyr416) (Fig. 7B), while the total Src (Fig. 7A) and the phosphorylation at Tyr527 (Fig. 7C) were unaltered. Conversely, the knock-down of NKAα3 increased the total Src (Fig. 7A) and ERK1/2 (Fig. 7F), but did not alter their phosphorylation (Fig. 7B, C, G). The abundance of the total or phosphorylated EGFR (Fig. 7D, E), ACC (Fig. 7H,I), and Akt (Fig. 7J) was not altered by knocking-down NKAα1 or NKAα3.

### Effect of Ouabain on Cell Viability and Energy Metabolism

**Fig. 8:**
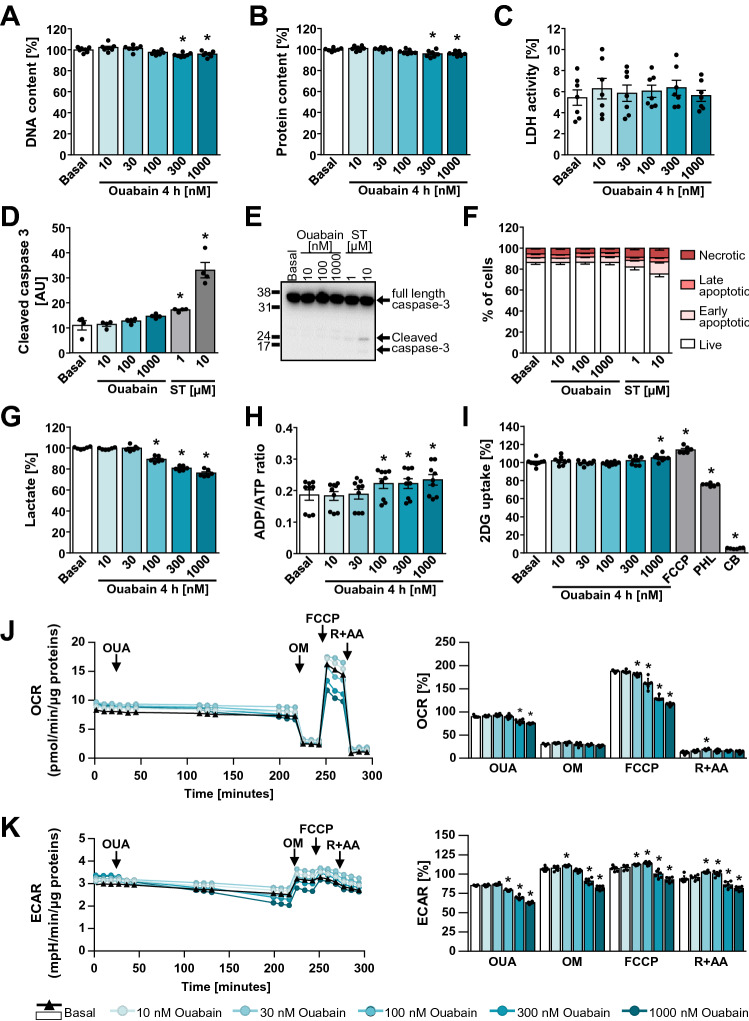
Metabolic effects of ouabain in HK-2 cells. **A**–**I** HK-2 cells were incubated in serum-free DMEM and treated with indicated concentrations of ouabain (OUA, 4 h), staurosporine (ST), phlorizin (PHL), or cytochalasin B (CB). DNA content (**A**), protein content (**B**), LDH activity (**C**), lactate production (**G**), and the ADP/ATP ratio (**H**) were measured using specific assays (see Methods). Immunoblotting (**D**, **E**) was used to estimate protein levels of full-length and cleaved caspase-3. Flow cytometry (**F**) was used to determine % of live, early apoptotic, late apoptotic, and necrotic cells. Glucose uptake (**I**) was evaluated by measuring the uptake of ^3^H-labelled 2-DG. DNA content, protein content, lactate production, and 2-DG uptake are expressed as % of basal. LDH activity is expressed as % of Triton X-100 treated group. **J**–**K** Oxygen consumption rate (OCR) (**J**) and extracellular acidification rate (ECAR) (**K**) were analysed with Seahorse XFe24 Analyzer. Cells were treated with ouabain or vehicle for 3½ h before the sequential addition of oligomycin (OM), FCCP, and a combination of rotenone and antimycin A (R + AA). The graphs on the left show the average absolute changes in OCR and ECAR during the course of experiment according to the treatment scheme. The graphs on the right show OCR and ECAR (average of three measurements) after 3½ h of ouabain treatment and after addition of OM, FCCP, and R + AA, expressed as % of basal (average of first three measurements before addition of ouabain). Results are means with SEM (two or three independent experiments, *n* = 6–9). *P < 0.05 vs. basal (unpaired one-way ANOVA, Dunnett’s test)

Ouabain was used in concentrations that are sufficient to suppress pumping activity of human NKAα1 (Wang et al. 2001), which might have caused disruption of ion homeostasis, reduction of cell viability and/or metabolic alterations. On the other hand, inhibition of NKA by ouabain might have led to ATP savings and improved energy status, which would tend to oppose AMPK activation. To assess these possibilities, HK-2 cells were treated with 10–1000 nM ouabain for 4 h (Fig. 8). Ouabain in high concentrations (300–1000 nM) somewhat reduced the content of DNA (Fig. 8A) and proteins (Fig. 8B), but did not increase release of lactate dehydrogenase (LDH) (Fig. 8C). As assessed by immunoblotting, caspase-3 was not activated by ouabain (Fig. 8D, E), indicating apoptosis was not induced. As estimated by flow cytometry, ouabain also did not increase the fraction of necrotic and apoptotic cells (Fig. 8F). These results demonstrated that 100 nM ouabain did not cause significant cell death.

Ouabain reduced the lactate production in dose-dependent manner (Fig. 8G), which indirectly suggested that the rate of glycolysis was reduced due to NKA inhibition and lower ATP consumption. However, treatment with ouabain increased the ADP:ATP ratio (Fig. 8H), which indicated that ouabain impaired the energy balance in HK-2 cells. To test whether ouabain altered glucose uptake, HK-2 cells were treated with ouabain (10–1000 nM), FCCP (a mitochondrial uncoupler), phlorizin (an inhibitor of Na^+^-coupled glucose transporters SGLT1 and 2), and cytochalasin B (a glucose transporter inhibitor) (Fig. 8I). Excepting the highest concentration (1000 nM) ouabain did not alter the uptake of 2-deoxy-glucose (2-DG). FCCP increased 2-DG uptake, while phlorizin had a modest suppressive effect, indicating SGLT transporters were active in these cells. Cytochalasin B almost completely suppressed the uptake of 2-DG.

To determine whether ouabain altered mitochondrial function, the oxygen consumption rate (OCR) (Fig. 8J) and extracellular acidification rate (ECAR) (Fig. 8K) were determined in HK-2 cells. Under basal conditions OCR was reduced by 300 and 1000 nM ouabain, while ECAR was reduced already by 100 nM ouabain, consistent with the reduced lactate production (Fig. 8G). While this indicated that glycolytic ATP production was more sensitive to ouabain than oxidative metabolism, 30–100 nM ouabain was sufficient to suppress the FCCP-stimulated (i.e. maximal) OCR, suggesting that mitochondrial function was highly sensitive to ouabain action.

## Discussion

A major finding of our study is that the EGF-stimulated phosphorylation of NKAα1 at Tyr10 was suppressed by two different AMPK activators and enhanced by the cardiotonic steroid ouabain. This finding highlights the phosphorylation of Tyr10 as a focal point of regulation by multiple signalling pathways. Moreover, it implicates a role for AMPK in regulation of tyrosine phosphorylation of NKA.

EGF was previously shown to increase tyrosine phosphorylation of NKAα1 in the proximal tubules (Feraille et al. 1997). Here we extend this pioneering result by showing that EGF stimulates the phosphorylation of Tyr10, which likely occurs as a consequence of the activation of the EGFR-Src pathway. First, inhibition of EGFR by gefitinib blocked the EGF-stimulated phosphorylation of Tyr10, demonstrating that activation of EGFR is absolutely required for this effect. Second, EGF could not increase phosphorylation of Tyr10 in the presence of Src kinase inhibitor PP2. Importantly, EGF increased the phosphorylation of EGFR despite PP2, indicating that activation of EGFR does not lead to Tyr10 phosphorylation if Src is inhibited. Src and Lyn, another member of the Src kinase family, were previously shown to phosphorylate NKAα at tyrosine residues under in vitro conditions (Al-Khalili et al. 2003; Bozulic et al. 2004a), while PP2 blocked the phosphorylation of Tyr10 in ocular ciliary epithelial cells (Shahidullah et al. 2014). Finally, unlike Src the activation of ERK1/2 did not seem to be functionally important for regulation of Tyr10 phosphorylation in HK-2 cells. Indeed, the phosphorylation of ERK1/2 was increased by EGF even in the presence of PP2, while A-769662, which suppressed Tyr10 phosphorylation activated ERK1/2. Taken together, all this points to a mechanism whereby EGF stimulates EGFR, which in turn phosphorylates and activates Src or another member of the Src family, thus leading to the phosphorylation of Tyr10.

Activation of AMPK by AICAR as well as A-769662 suppressed the EGF-stimulated phosphorylation of Tyr10. The phosphorylation of EGFR was suppressed by both AMPK activators, supporting the notion that activation of EGFR is important for the phosphorylation of Tyr10 by EGF. Consistent with our results, suppression of EGFR by AMPK was observed in cultured cancer cells (Jhaveri et al. 2015). In contrast, A-769662 increased the (activating) phosphorylation of Src at Tyr416 in HK-2 cells in the presence and absence of EGF. Despite an apparent activation of Src, the EGF-stimulated phosphorylation of Tyr10 was markedly suppressed by A-769662, which may seem to contradict a role for Src as Tyr10 kinase. However, on the other hand, A-769662 did not alter the Tyr10 phosphorylation in the absence of EGF despite a marked increase in the phosphorylation of Src. These results suggest that activation of Src without activation of EGFR does not lead to phosphorylation of Tyr10.

One explanation for the dissociation between phosphorylation of Src and Tyr10 could be that A-769662 activates a pool of Src that is not co-localized with EGFR and NKA. Indeed, localization of non-receptor tyrosine kinases, such as Src, in the vicinity of their targets is important for their substrate specificity (de Diesbach et al. 2008; Miller 2003). Another explanation could be that the activation of AMPK stimulates tyrosine phosphatases, which oppose phosphorylation of Tyr10 by Src. This explanation is not unlikely, since AMPK was previously shown to regulate the phosphorylation of Ser18 via serine/threonine phosphatase PP2A (Benziane et al. 2012). In summary, our data suggest that AMPK blocks the phosphorylation of Tyr10 by suppressing activity of EGFR, but additional mechanisms, such as involvement of phosphatases, cannot be excluded.

PP2, a selective Src inhibitor, also inhibits EGFR, albeit significantly less potently (IC_50_ of 480 nM for EGFR versus 5 nM for Src) (Hanke et al. 1996). It can therefore be speculated that 20 μM PP2 at least partially inhibited EGFR, which would be consistent with a lower phosphorylation of Tyr1173 after the stimulation with EGF. On the other hand, Src is a downstream target of EGFR and, once activated by it, Src can in turn phosphorylate and activate EGFR in a positive feedback manner (Biscardi et al. 1999). Thus, inhibition of Src by PP2 could also indirectly reduce the EGF-induced activation (phosphorylation) of EGFR. Although both mechanisms may have been involved, PP2 did not prevent the EGF-induced phosphorylation of EGFR, while abrogating the EGF-induced Tyr10 phosphorylation of NKAα1. This result suggests that the phosphorylation of Tyr10 requires an increase in the Src activity and cannot be achieved by activation of EGFR alone.

However, it also needs to be considered that alterations in the phosphorylation of Src are not always a reliable marker of its activity, so results of immunoblots in the presence of inhibitors of Src, which bind to it directly, should be interpreted cautiously. Even well-established inhibitors, such as PP2, do not always result in predictable changes in the phosphorylation of Src. Intuitively, one could expect that pharmacological inhibition of Src results in a reduced phosphorylation of the activation site Tyr416 (aka Tyr418) and an increased phosphorylation of the inhibitory site Tyr527 (aka Tyr529). In direct contrast, PP2 (in concentrations that abolish Src activity) may enhance the phosphorylation of Src at Tyr416, induced by exogenous stimuli, such as bombesin and angiotensin II (Wu et al. 2005). A similar mechanism might explain why genistein enhanced the EGF-stimulated phosphorylation of Src at Tyr416 in our experiments. Further, PP2 alone does not necessarily reduce the basal phosphorylation of Src at Tyr416 or even increases it (Wu et al. 2005). This means that the phosphorylation of Tyr416 is not a reliable marker of the Src activity in the presence of PP2. In addition, PP2 does not enhance the phosphorylation of Src at the inhibitory site Tyr527, but may even promote its dephosphorylation (Wu et al. 2005), which again highlights the challenge of assessing activity of kinases from their phosphorylation status in the presence of pharmacological inhibitors.

Clearly, pharmacological inhibition of Src does not result in simple alterations of its phosphorylation status, from which activity of Src could be inferred under all conditions. Similarly, high glucose concentrations or phorbol 12-myristate 13-acetate increased the phosphorylation of Ser and Thr residues and decreased the NKA activity, while insulin increased the phosphorylation of Ser, Thr, and Tyr residues and increased the NKA activity in rat skeletal muscle (Chibalin et al. 2001). Thus, an increase in Ser and Thr phosphorylation was associated both with an increase and a decrease of the NKA activity, which demonstrates that the final physiological effect is likely dependent on the phosphorylation pattern of all functionally important phosphosites and possibly also on the phosphorylation status of proteins that are involved in regulation of NKA trafficking and/or activity.

The physiological role of AMPK is frequently assessed by using pharmacological AMPK activators. The challenge of this approach is that pharmacological compounds have off-target effects that are not directly dependent on AMPK activation, which makes interpretation of results challenging. This complexity can be appreciated also in our results, which clearly showed that activation of AMPK by AICAR and A-769662 did not produce the same signalling responses. For instance, the phosphorylation of Src as well as ERK1/2 was markedly increased by A-769662. In contrast, AICAR did not alter the phosphorylation of Src, but it suppressed the phosphorylation of ERK1/2 under basal conditions (in the absence of EGF). Despite these differences both AMPK activators decreased the phosphorylation of EGFR as well as NKAα1 at Tyr10, which strengthens the argument that AMPK plays a role in regulation of Tyr10 phosphorylation via inhibition of the EGFR signalling.

Ouabain enhanced the EGF-stimulated phosphorylation of Tyr10 although it had no effect when used alone. Importantly, EGF action on Tyr10 was enhanced although phosphorylation of EGFR and Src were similar between the EGF and the combined (EGF and ouabain) treatment. While the underlying mechanism was not dissected it seems clear that ouabain did not promote the phosphorylation of Tyr10 by augmenting the activation of EGFR-Src pathway. We can therefore speculate that ouabain might have induced conformational changes in NKAα1 that made Tyr10 more accessible to phosphorylation by Src. Alternatively, ouabain might have enhanced the association between NKA and Src. If ouabain increased the interaction between NKA and Src via either of these two mechanisms, the pool of activated Src molecules may have been more efficient at phosphorylating Tyr10. This hypothesis is consistent with the above stated idea that A-769662 activated a pool of Src that was not close to NKA. Notably, ouabain increased interaction between Src and NKAα1 and NKAα2 in isolated skeletal muscle (Kotova et al. 2006b), which supports the idea that ouabain might increase phosphorylation of Tyr10 without affecting the activity of EGFR and other upstream signalling pathways.

The EGFR/Src/ERK1/2 signalling pathway was traditionally considered to be a particularly important link between ouabain and its intracellular effects. However, its universal significance as well as the underlying molecular mechanisms that link NKA to this pathway have recently been questioned (Askari 2019a, b). While the present study was not designed to test the importance of this pathway for ouabain actions, our results do seem to suggest that relatively short treatment with ouabain did not induce marked signalling responses in HK-2 cells. This observation is consistent with our recent study in cultured human skeletal muscle cells, in which we showed that most signalling responses occurred during prolonged ouabain treatments, which suggested that they had been mediated by alterations in intracellular concentrations of Na^+^ and K^+^ (Pirkmajer et al. 2020). Moreover, in HK-2 cells ouabain somewhat suppressed the phosphorylation of ERK1/2 and induced a minor increase in the inhibitory phosphorylation of Src (Tyr527), which again shows that ouabain did not acutely activate the EGFR/Src/ERK1/2 pathway in HK-2 cells. Gene silencing of NKAα1 or NKAα3 also did not induce major changes in intracellular signalling in non-stimulated cells. Nevertheless, the abundance of the phosphorylated Src (Tyr416) in the NKAα1-deficient cells as well as the total Src and ERK1/2 in the NKAα3-deficient cells was altered. Thus, although ouabain did not acutely activate the EGFR/Src/ERK1/2 pathway in HK-2 cells, our results are compatible with existence of a functional link between ouabain, NKA, and the EGFR/Src/ERK1/2 signalling.

Ouabain had no effect on the ATP production in isolated mitochondria (Kajikawa et al. 2002), but it can alter mitochondrial respiration via several indirect mechanisms. First, inhibition of NKA reduces the cellular demand for ATP, thus leading to reduced OCR, although this effect would depend on the cell type because fractional use of ATP by NKA varies widely between different cells and tissues (Clausen et al. 1991; Rolfe and Brown 1997). However, a reduction in ATP consumption did not seem to be a major mechanism in HK-2 cells because ouabain increased the ADP:ATP ratio, which indicated that the ATP production was impaired rather than reduced as a consequence of a lower demand for ATP. That ouabain impairs the ATP production was shown also in isolated pancreatic islets (Kajikawa et al. 2002) and cultured cancer cells (Shen et al. 2020). This impairment may result from inhibition of ion transport, which leads to osmotic imbalance, water uptake, and mitochondrial swelling (Leonard et al. 2015). On the other hand, while ouabain reduced the glucose oxidation in isolated skeletal muscle, inhibition of Src family kinases by PP2 blocked this effect (Kotova et al. 2006b), which not only shows that inhibition of ion transport per se is not always sufficient to alter mitochondrial function, but also highlights the link between ouabain-induced signalling via NKA and regulation of energy metabolism. Consistent with this notion, NKAα1 and Src were recently shown to play an important role in regulating mitochondrial respiration (Kutz et al. 2021).

In addition, ouabain was previously shown to increase glycogen synthesis in isolated skeletal muscles as well as cultured skeletal muscle cells (Clausen 1966; Kotova et al. 2006a, 2006b). Interestingly, in our current study a reduction in the lactate production, ECAR, and OCR as well as the FCCP-stimulated maximal OCR was not paralleled by alterations in the uptake of 2-DG. While we did not measure the glucose oxidation, these results showed that the net glucose uptake was unaltered, while glycolysis and OCR were reduced. Our results are therefore compatible with the possibility that ouabain may have redirected glucose towards glycogen synthesis in HK-2 cells.

Here we did not examine the functional consequences of the Tyr10 phosphorylation, but these may theoretically include modulation of NKA kinetics, protein–protein interactions with signalling proteins or NKA regulators, such as FXYD proteins, or NKA trafficking between the plasma membrane and the intracellular compartment. The ultimate effect of the tyrosine phosphorylation of NKAα1 is unclear since the studies which examined the functional significance of unspecific tyrosine phosphorylation or phosphorylation of Tyr10 did not produce consistent results. For instance, insulin and EGF increased tyrosine phosphorylation of NKAα1 and activity of NKA in rat proximal tubules (Feraille et al. 1999, 1997). In line with these findings, bromocriptine, a dopaminergic agonist, increased the membrane abundance of NKAα1, Tyr phosphorylation of NKAα1, and NKA activity in cultured rat proximal tubular cells (Narkar et al. 2002a, 2002b). Conversely, tyrosine phosphorylation of NKAα1 in epithelial cells of the lens suppressed NKA activity (Bozulic et al. 2004a, 2004b). Moreover, incubation of NKA isolated from the porcine kidney with dephostatin, an inhibitor of protein tyrosine phosphatases, reduced the NKA activity (El-Beialy et al. 2010). Similarly, in the rat diaphragm muscle eplerenone, an aldosterone receptor antagonist, reduced phosphorylation of Tyr10, while NKA activity was increased, suggesting Tyr10 is an inhibitory site (Breitenbach et al. 2016). Activation of Src family kinases and an increase in phosphorylation of Tyr10 in ocular ciliary epithelial cells was also suggested to mediate inhibition of NKA by nitric oxide (Shahidullah et al. 2014). It therefore appears that the functional consequences of the Try10 phosphorylation might differ across tissues.

## Conclusions

In conclusion, AMPK activation suppressed the EGF-stimulated phosphorylation of Tyr10 in HK-2 cells, while NKA inhibitor ouabain promoted it. AMPK likely exerted this effect by suppressing the EGFR-Src pathway, but activation of tyrosine phosphatases, which dephosphorylate Tyr10 cannot be excluded. The mechanism by which ouabain enhanced the EGF-stimulated Tyr10 phosphorylation did not seem to involve altered signalling via EGFR or Src. Collectively, our results suggest a new mechanism by which AMPK regulates NKA, while highlighting a link between regulation of energy metabolism and NKA-mediated ion transport.

## Data Availability

Original blots and PCR data files are available in the supplement to the manuscript.
